# PI-RADS 2.1 is valid for detecting prostate cancer in bpMRI in an Irish population

**DOI:** 10.1007/s11845-026-04469-x

**Published:** 2026-06-18

**Authors:** Patrick A. Williams, Nesa Samuel Karunadhas, Niamh Moynagh, Mohammed Janquli, Mutaz Nur, Pádraig J. Daly, Ciarán E. Redmond

**Affiliations:** 1 Faculty of Radiology and Radiation Oncology, Royal College of Surgeons in Ireland, Dublin, Ireland; 2 Department of Radiology, University Hospital Waterford, Waterford, Ireland; 3 Department of Pathology, University Hospital Waterford, Waterford, Ireland; 4 Department of Surgical Affairs, Royal College of Surgeons in Ireland, Dublin, Ireland; 5 Department of Urology, University Hospital Waterford, Waterford, Ireland

**Keywords:** Prostate cancer, Prostate MRI, Biparametric MRI, PI-RADS 2.1

## Abstract

**Background:**

Magnetic resonance imaging (MR) of the prostate is an important part of the diagnostic pathway for patients with suspected prostate cancer. Current practice in many Irish centres is to perform biparametric MRI (bpMRI) incorporating T2- and diffusion-weighted imaging, and interpret it using the latest version of the American College of Radiology Prostate Imaging Reporting and Data System (PI-RADS), version 2.1. No study has yet reported on the use of bp-MRI and PI-RADS 2.1 in the investigation of prostate cancer investigation in the Irish healthcare setting.

**Aims:**

We aimed to review one year of practice at our regional tertiary prostate cancer referral centre and compare diagnostic yield with international standards.

**Methods:**

We identified all patients who had undergone prostate biopsy at our institution in 2023 and extracted pre-biopsy MRI results for each patient, comparing MRI results with biopsy reports.

**Results:**

We identified 328 patients in the study period who underwent prostate biopsy and pre-biopsy MRI. 182 of these had a pathological diagnosis of clinically significant prostate cancer (csPCa), defined as Gleason score of 7 or higher. Of these, 167 (92%) had a PI-RADS score of 3 or above, meaning at least equivocal for the presence of csPCA. Cancer detection rates were 31%, 54% and 96% for PI-RADS three, four and five lesions respectively. We demonstrated a sensitivity of 92% and specificity of 49% for detection of csPCa, closely aligned with international datasets.

**Conclusions:**

The use and performance of bpMRI and PI-RADS 2.1 at our Irish tertiary urological centre is in line with international datasets. These have a key role in the diagnostic pathway of prostate cancer in Ireland.

## Introduction

 Prostate cancer poses a diagnostic and therapeutic challenge for clinicians. While it is the third most common cancer and the fifth leading cause of death in males, it exists on a clinical spectrum from indolent to highly aggressive [1]. Guidelines recommend screening for prostate cancer using clinical history, digital rectal examination and testing serum levels of Prostate Specific Antigen (PSA) [2]. Subsequent investigation with Magnetic Resonance Imaging (MRI) has become standard practice in our institution and beyond due to evidence that it increases the detection of clinically significant prostate cancer and reduces the diagnosis of clinically insignificant disease [3, 4]. By obtaining an MRI scan prior to deciding whether to proceed to biopsy, we aim to diagnose and treat those who have clinically significant disease while avoiding unnecessary investigations and treatment in those whose disease is insignificant.

Prostate cancer is graded using histological analysis of biopsy specimens. Clinically significant prostate cancer (csPCa) is distinguished from clinically insignificant disease by classification using the Gleason scoring system as recommended by the International Society of Urothelial Pathology [5, 6]. This system records patterns of cellular proliferation on a scale from three to five, with higher scores indicating higher-risk disease. The Gleason score of a biopsy sample is expressed as a sum of the two most common patterns in a biopsy sample, for example, 4 + 3=7 or 4 + 8=8. A Gleason score of 7 or above is the widely accepted threshold for clinically significant cancer [6].

Prostate biopsies are commonly performed via transrectal and transperineal approaches. Many institutions, ours included, favour the transperineal approach due to reduced infection rates and increased detection rates of anterior lesions [7–9].

The prostate imaging-reporting and data (PI-RADS) system developed by the American College of Radiologists is the widely accepted standard for quantitative analysis of prostate MRI. Under this system, lesions are classified on a scale from one (clinically significant cancer is highly unlikely to be present) to five (clinically significant cancer is highly likely to be present). A significant lesion is defined to be one with a PI-RADS score of three (the presence of clinically significant cancer is equivocal) or higher [10].

The latest iteration of PI-RADS, version 2.1, was published in 2019 and provides for multi-parametric MRI (mpMRI) with T2-weighted, diffusion-weighted and contrast-enhanced images of the prostate in multiple planes [10]. Since the publication of this standard, several authors have advocated a bi-parametric MRI protocol (bpMRI) where contrast-enhanced imaging is omitted to reduce acquisition time and avert the potential adverse events of intravenous contrast agents. Studies have found that bp- and mpMRI have comparable accuracy in detecting significant lesions [11–14]. 

PI-RADS 2.1 has been in use at our institution since 2020, and we routinely use bpMRI as part of our diagnostic pathway. This study is a retrospective review of one year of our practice. We aimed to assess the diagnostic accuracy of bpMRI compared with biopsy in our institution to evaluate our practice and provide the first published dataset of PI-RADS 2.1 in an Irish population.

## Materials and methods

### Participant selection

We used our hospital Laboratory Information Management System (LIMS) to identify every patient who underwent prostate biopsy in our institution in 2023. We then reviewed prostate MRI studies for each patient and compared radiology reports with pathology reports, as documented at MDT discussion.

We excluded patients whose scans had not demonstrated evidence of clinically significant cancer (PI-RADS 1 or 2) as these patients only proceeded to biopsy if they had other risk factors, thus PI-RADS was not being used as the primary means of risk stratification.

### Prostate MRI technique

We included prostate MRI studies performed on four different 1.5T MRI scanners across three hospitals that refer to our centre. In all cases, T2-weighted images were obtained in axial, coronal and sagittal planes, and axial DWI images were used to generate ADC maps. We did not assess for dynamic contrast enhancement. Images were interpreted by five radiologists with a minimum of seven years of experience, according to PI-RADS 2.1 standards.

### Prostate biopsy technique

All biopsies were carried out by a consultant urologist, urology trainee or advanced nurse practitioner using a transperineal ultrasound-guided approach under general, spinal or regional anaesthesia. Patients lie in the lithotomy position with scrotal elevation and digital rectal examination is performed to evaluate prostate size, consistency and clinical T-stage. The perineum is prepped with an antiseptic solution and a lubricated ultrasound probe is inserted into the rectum. The prostate gland can be visualised in axial and sagittal planes. The prostate is split into right and left lobes with biopsies taken from apex to base and anterior to posterior using a free hand technique. Systematic biopsies are taken with additional target biopsies based on MRI findings. Tissue samples are placed in formalin and labelled according to gland region prior to being sent to the histopathology lab.

### Prostate biopsy analysis

All core biopsies were processed and reported according to the Royal College of Pathologists (RCPath) dataset for prostate cancer [15]. Reports included the Gleason score and the International Society of Urological Pathology (ISUP) grade group. Other factors noted in the reports included histological type of prostate cancer, number of positive cores positive on each side and in other specified locations, total number of positive cores and at least one measure of tumour extent (total percentage of cancer in all cores, greatest percentage of cancer in one core or longest length of tumour in one core) [6]. All malignant cases were independently reviewed and authorised by two pathologists.

### Statistical analysis

Statistical analysis was performed in Microsoft Excel 2021 (Microsoft, Redmond, Washington, USA). We calculated sensitivity and specificity using a 2 × 2 contingency table with biopsy results stratified as Gleason score of 6 or below or Gleason score of 7 or higher, and MRI results stratified by PI-RADS score of 2 or lower and PI-RADS score of 3 or higher. We calculated cancer detection rates (CDRs) for each PI-RADS score by stratifying the biopsy results as above for each PI-RADS score of 3–5.

## Results

### Demographics and patient characteristics

We identified 351 patients who underwent prostate biopsy in our institution in 2023. All patients were males between the age of 40 and 89 with a median age of 66. Of 351 patients, 10 did not have an MRI due to MRI-incompatible implants or other technical reasons, and 13 underwent MRI but did not have PI-RADS scores assigned as their studies were non-diagnostic.

Therefore, 328 patients had Gleason scores and PI-RADS scores to enable comparison. Of these 161 (49%) had clinically significant prostate cancer (Gleason score of 7 or above).

### Lesion detection

182 of 328 patients (55%) had clinically significant prostate cancer on prostate biopsy. Of these, 167 (92%) had a PI-RADS 2.1 score of 3 or above.

The sensitivity of bpMRI for the detection of csPCa was 92%, and the specificity was 49%. The positive predictive value of bpMRI was 69%, and the negative predictive value was 83%.

### Lesion detection by PI-RADS score

Of 48 patients with a PI-RADS 3 lesion on bpMRI, 15 (31%) had csPCa on biopsy. Of 81 patients with a PI-RADS 4 lesion, 44 (54%) had csPCa. Of 113 patients with a PI-RADS 5 lesion on MRI, 108 (96%) had csPCa on biopsy (Table 1).

**Table 1:** csPCa rates stratified by PI-RADS MRI score

PI-RADS score	csPCA (by Gleason score)		Total	Cancer detection rate (CDR)
	Yes	No		
3	15	33	48	31%
4	44	37	81	54%
5	108	5	113	96%

13 patients with PI-RADS 5 lesions on initial MRI had benign biopsies. Of these 11 underwent repeat biopsy after MDT discussion. Six of these patients had csPCa on repeat biopsies, and five had a second benign biopsy. Of the two patients who did not undergo repeat biopsy, one underwent repeat MRI due to technical limitations on the initial study. His lesion was downgraded to PI-RADS 2 on the second MRI. The second patient’s MRI was downgraded to PI-RADS 2 after consensus review at MDT.

## Discussion

Our data shows PI-RADS 2.1 has a high sensitivity for csPCa on prostate biopsy in our cohort and rates of csPCa increase with higher PI-RADS scores. To validate our data, we compared to a systematic review performed by Oerther et al. in 2024 which was intended for use to benchmark local diagnostic practice. This study analysed 70 primary studies which included a total of 13,330 patients [16]. 

When assessing the diagnostic accuracy of PI-RADS 3 or greater at the patient level, our practice performed very similarly to the international benchmark. Our data showed sensitivity of 92% and specificity of 49% compared to 96% and 45%, respectively in the benchmark dataset. (Table 2).

Regarding cancer detection rate (CDR), the rate of csPCa detected for each PI-RADS score, our results for PI-RADS 4 and 5 lesions were closely aligned with the international benchmark. We found CDRs of 54% and 96% for PI-RADS scores of 4 and 5 respectively, compared to 54% and 84% in the benchmark dataset.

Our CDR for PI-RADS 3 lesions was higher than the benchmark, at 31% versus 19%. This may be accounted for by the routine use of targeted biopsies in addition to standard template-guided approaches in our centre when PIRADs 3 lesions are detected. The studies included in the benchmark dataset were heterogeneous in biopsy technique with some using only a template approach. (16)

Our results are in line with the international benchmark, and thus we have validated the use of PI-RADS 2.1 in an Irish cohort. In doing so, we have also validated the Irish National Cancer Control Programme (NCCP) clinical guideline for the diagnosis of prostate cancer, which is in use in our centre and all other large Irish cancer centres, in an Irish cohort. This guideline recommends pre-biopsy MRI interpreted using the latest version of PI-RADS in all patients in whom it is practicable [17]. As the first validation of PI-RADS 2.1 in an Irish cohort, the present study validates this guideline recommendation.

We omit dynamic contrast-enhanced sequences from our prostate MRI protocol in our centre. Non-contrast studies have shorter acquisition times, increased tolerability for patients, reduced costs and are in keeping with the principle of reducing use of gadolinium-based contrast agents (GBCAs) due to environmental concerns [18]. There is a body of literature comparing the diagnostic yield of non-contrast bpMRI studies to traditional mpMRI studies, which shows generally similar rates of cancer detection, PPv and NPV for bpMRI and mpMRI [11–14, 16].

Our study adds to this literature by demonstrating equivalent performance bpMRI in our population to the benchmark study in which most included studies employed mpMRI. of in which most studies employed mpMRI.

The chief limitation of our study is that it involves only one centre and one year of practice. While we have validated the PI-RADS 2.1 standards in an Irish cohort, this study is not a comprehensive review of the use of prostate MRI in Ireland, and data from other centres will be useful to enable benchmarking within Ireland.

A second limitation of our study is that, while bpMRI has been studied widely and found to be equivalent to mpMRI, the benchmarking dataset we used contained studies using mpMRI. Given the above-mentioned equivalence between the methods, this should not impact the comparison. However, given the increasing use of bpMRI in many centres, a future benchmarking project would benefit from providing separate datasets for bpMRI and mpMRI. This would ensure that the two techniques remain comparable as MRI protocols and reporting standards develop in the future.

**Table 2:** Sensitivity and specificity of PI-RADS 2.1 in our dataset compared to a recent systematic review

	Oerther et al, 2024		Our data	
	Sensitivity	Specificity	Sensitivity	Specificity
PI-RADS >/=3	96%	45%	92%	49%

## Conclusions

Biparametric MRI interpreted with PI-RADS 2.1 is valid for pre-biopsy assessment of patients suspected to have prostate cancer in Ireland. Our results are in line with internationally published systematic reviews. This study adds to the increasing literature validating bpMI for detection of csPCA.
